# Validation of image-derived input function using a long axial field of view PET/CT scanner for two different tracers

**DOI:** 10.1186/s40658-024-00628-0

**Published:** 2024-03-13

**Authors:** Xavier Palard-Novello, Denise Visser, Nelleke Tolboom, Charlotte L. C. Smith, Gerben Zwezerijnen, Elsmarieke van de Giessen, Marijke E. den Hollander, Frederik Barkhof, Albert D. Windhorst, Bart NM van Berckel, Ronald Boellaard, Maqsood Yaqub

**Affiliations:** 1 Univ Rennes, CLCC Eugène Marquis, INSERM, LTSI - UMR 1099, Rennes, France; 2 Amsterdam University Medical Center, Vrije Universiteit Amsterdam, Amsterdam, The Netherlands; 3 Amsterdam Neuroscience, Brain Imaging, Amsterdam, The Netherlands; 4 University Medical Center Utrecht, Utrecht, The Netherlands; 5 Cancer Center Amsterdam, Imaging and Biomarkers, Amsterdam, The Netherlands; 6 Queen Square Institute of Neurology and Centre for Medical Image Computing, University College London, London, UK

**Keywords:** [^18^F]DPA-714, [^18^F]FDG, LAFOV PET/CT, IDIF

## Abstract

**Background:**

Accurate image-derived input function (IDIF) from highly sensitive large axial field of view (LAFOV) PET/CT scanners could avoid the need of invasive blood sampling for kinetic modelling. The aim is to validate the use of IDIF for two kinds of tracers, 3 different IDIF locations and 9 different reconstruction settings.

**Methods:**

Eight [^18^F]FDG and 10 [^18^F]DPA-714 scans were acquired respectively during 70 and 60 min on the Vision Quadra PET/CT system. PET images were reconstructed using various reconstruction settings. IDIFs were taken from ascending aorta (AA), descending aorta (DA), and left ventricular cavity (LV). The calibration factor (CF) extracted from the comparison between the IDIFs and the manual blood samples as reference was used for IDIFs accuracy and precision assessment. To illustrate the effect of various calibrated-IDIFs on Patlak linearization for [^18^F]FDG and Logan linearization for [^18^F]DPA-714, the same target time-activity curves were applied for each calibrated-IDIF.

**Results:**

For [^18^F]FDG, the accuracy and precision of the IDIFs were high (mean CF ≥ 0.82, SD ≤ 0.06). Compared to the striatum influx (*K*_*i*_) extracted using calibrated AA IDIF with the updated European Association of Nuclear Medicine Research Ltd. standard reconstruction (EARL2), *K*_*i*_ mean differences were < 2% using the other calibrated IDIFs. For [^18^F]DPA714, high accuracy of the IDIFs was observed (mean CF ≥ 0.86) except using absolute scatter correction, DA and LV (respectively mean CF = 0.68, 0.47 and 0.44). However, the precision of the AA IDIFs was low (SD ≥ 0.10). Compared to the distribution volume (*V*_*T*_) in a frontal region obtained using calibrated continuous arterial sampler input function as reference, *V*_*T*_ mean differences were small using calibrated AA IDIFs (for example *V*_*T*_ mean difference = -5.3% using EARL2), but higher using calibrated DA and LV IDIFs (respectively + 12.5% and + 19.1%).

**Conclusions:**

For [^18^F]FDG, IDIF do not need calibration against manual blood samples. For [^18^F]DPA-714, AA IDIF can replace continuous arterial sampling for simplified kinetic quantification but only with calibration against arterial blood samples. The accuracy and precision of IDIF from LAFOV PET/CT system depend on tracer, reconstruction settings and IDIF VOI locations, warranting careful optimization.

## Introduction

The standardized uptake value (SUV) is a widely used semi-quantitative metric referring to the measurement of tracer uptake based on a single-time-point [1]. However, even for the most widespread tracer 18F-fluorodeoxyglucose ([^18^F]FDG), there are still concerns regarding its accuracy for quantification of uptake in lesions, mostly due to the variation of the availability of [^18^F]FDG in the blood, which is poorly accounted in the SUV calculation [2].

Dynamic PET imaging offers information about the tracer concentration over time and enables the extraction of kinetic parameters such as the net influx rate (*K*_*i*_) for tracers with irreversible binding or the distribution volume (*V*_*T*_) for tracers with reversible binding. Compared to SUV, studies have shown that *K*_*i*_ is a more robust and accurate index of glucose metabolic rate for [^18^F]FDG [3, 4]. However, an accurate arterial input function is needed to determine this parameter. The reference method for extracting an accurate arterial input function is the use of a continuous arterial blood sampler and calibrated with several manual arterial samples [5] which is invasive and challenging as arterial blood sampling is laborious and could have some adverse effects [6, 7]. For [^18^F]FDG, studies using short axial field-of-view (FOV) showed previously that image derived input function (IDIF) from aorta or left ventricle can replace continuous arterial blood sampler [8–10] and IDIF from aorta or left ventricle can replace manual blood samples for kinetic modelling [11]. However, the short axial FOV (∼ 20 cm) PET scanner limits the ability to obtain accurate IDIF because aorta or left ventricle can be located outside the FOV. Due to the limited spatial resolution of the PET scanners, measured activity from small arteries for brain dynamic scans (carotids) or pelvic dynamic scans (iliac or femoral arteries) are affected to spillover and partial-volume effects [12]. Some authors developed correction methods to mitigate the impact of these effects on quantification [12–17]. However, application of these methods is scarce. Moreover, in order to perform a whole-body simplified kinetic quantification using a short axial FOV PET system, Karakatsanis et al. developed a method which starts with a single dynamic cardiac-bed position acquisition followed by multiple whole-body acquisitions [18]. However, this method allows only low temporal sampling due to the iterative bed motion in the PET system.

Long axial field-of-view (LAFOV) (> 100 cm) PET scanner may overcome these limitations because major vessels and distant lesions are included in the FOV during the total dynamic acquisition time. LAFOV PET scanner offers up to a 3 times increase in peak axial sensitivity [19], leading to lower image noise and higher temporal sampling, opening up the possibility of better characterization of whole-body pathologies. However, extension of axial field-of-view increases the rates of random and scatter coincidences that could lead to quantification bias. To the best of our knowledge, no validation of using IDIF from the LAFOV PET scanner Biograph Vision Quadra with manual blood samples has been done yet, especially for [^18^F]FDG which is currently the most widely used radiotracer in clinical PET imaging. Regarding the location of choice for defining IDIF, ascending aorta (AA), descending aorta (DA) or left ventricular cavity (LV) are the most widely used regardless of the tracer applied. However, the IDIF location could impact the accuracy of IDIF and affect the quantification of kinetic parameters [8, 11]. Furthermore, the use of different reconstruction settings (matrix size, post-reconstruction filtering, scatter correction method or number of iterations/subsets) could potentially modify the time-activity curve of IDIF [20].

The aim of this study is to validate the use of IDIF for two kinds of tracers ([^18^F]FDG for irreversible uptake and [^18^F]DPA-714 for reversible uptake), 3 different IDIF VOI locations and 9 different reconstruction settings with a LAFOV PET/CT system.

## Materials and methods

### Study population

Between November 2022 and July 2023, 8 consecutive patients with [^18^F]FDG injection from a prospective study including patients with various diseases (TBPETCT001 study) and 10 consecutive patients with post COVID syndrome with [^18^F]DPA-714 injection from the VeCosCO study [21] scanned on the Siemens Biograph Vision Quadra at the Amsterdam UMC, location VUmc (the Netherlands) were included. Informed consent was obtained for all patients. These studies were approved by the Medical Ethics Review Committee of the Amsterdam UMC. The patients with [^18^F]DPA-714 injection were all high affinity binders based on TSPO genotype analysis.

### PET/CT data

Patients received 241 ± 61 MBq of [^18^F]FDG and 222 ± 27 MBq of [^18^F]DPA-714. All PET scans were performed after a low dose CT scan acquired without contrast agents. Acquisition of list-mode PET data started immediately after injection of the tracer. For [^18^F]FDG PET, the 70-min dynamic imaging data were binned into 20 frames (1 × 15 s, 3 × 5 s, 3 × 10 s, 4 × 60 s, 2 × 150 s, 2 × 300 s, 5 × 600 s). For [^18^F]DPA-714 PET, the 60-min dynamic imaging data were binned into 19 frames (1 × 15 s, 3 × 5 s, 3 × 10 s, 4 × 60 s, 2 × 150 s, 2 × 300 s, 4 × 600 s). For each scan, 9 different PET reconstructions were done using a dedicated offline reconstruction workstation (E7, Siemens Healthineers, Knoxville, TN, USA). All of the reconstructions shared the following settings: PSF + TOF OSEM algorithm, 5 subsets, decay correction, scatter correction and CT-based attenuation correction. The voxel size in the z direction was CT-matched (2 mm) or not CT-matched (1.65 mm). For each scan, this value was identical between reconstructions. For the scatter correction, relative scaling of the scatter correction is the default recommended option, but the use of absolute scatter scaling was explored as well. The other settings for the 9 reconstructions are detailed in Table 1.

**Table 1:** Reconstruction settings

	Maximum Ring Difference (MRD)	Iterations (it)	Matrix (mat)	Scatter correction	Gaussian post-filtering (4-mm FWHM)
EARL2	85	4	220 × 220	Relative	Yes
EARL2_MRD322	322	4	220 × 220	Relative	Yes
it8	85	8	220 × 220	Relative	Yes
it4_nogauss	85	4	220 × 220	Relative	No
it6_nogauss	85	6	220 × 220	Relative	No
it8_nogauss	85	8	220 × 220	Relative	No
it8_nogauss_mat440	85	8	440 × 440	Relative	No
it10_nogauss	85	10	220 × 220	Relative	No
it10_nogauss_abs	85	10	220 × 220	Absolute (abs)	No

### IDIF validation using calibration assessment with manual blood samples

For all patients, IDIFs were taken from a 2 × 2 (with matrix 220 × 220) or 4 × 4 (with matrix 440 × 440) voxels region placed centrally on five adjacent slices in the DA and LV on the updated European Association of Nuclear Medicine Research Ltd. standard reconstruction (EARL2) [22] used as reference and in the AA on all of the reconstructions (11 IDIFs in total: 9 derived from AA with different reconstruction settings, 1 IDIF from DA with EARL2 reconstruction and 1 IDIF from LV with EARL 2 reconstruction) using the ACCURATE software tool (developed in IDLversion 8.4 (Harris Geospatial Solutions, Bloomfield, USA)) [23]. For patients with [^18^F]FDG injection, 3 manual venous samples were used for calibration of the 11 different IDIFs (35, 45 and 60 min post-injection because the arterio-venous [^18^F]FDG concentration equilibrium is reached around 40 min post-injection [24]). For patients with [^18^F]DPA-714 injection, 4 manual arterial samples (at 20, 40, 60 and 75 min post-injection) were used for calibration of the 11 different IDIFs and for the input function obtained from an arterial continuous sampler (Comecer, Joure, the Netherlands). The calibration factor (CF) was the mean of the ratio between the whole blood activity from the manual samples and the whole blood activity from the IDIF (or the input function from the arterial continuous sampler for [^18^F]DPA-714) at the corresponding time points [12]. The CF was used for IDIFs accuracy and precision assessment.

### Uptake ratio between organs and aorta

In order to quantify the uptake for each tracer in the main organs close to the aorta which may affect the accuracy and the precision of IDIF, a VOI was manually drawn in the liver, lung, myocardium and spleen on the EARL2 reconstruction. The standardized uptake value normalized to body weight (SUVbw) for each organs VOI and AA was based on 50–60 min post-injection time interval.

### Illustration of the impact of different calibrated-IDIFs using kinetic modelling

#### Blood processing

For [^18^F]FDG, calibrated IDIFs were corrected for plasma to whole-blood ratios to obtain plasma input functions. The calibrated whole-blood curve was multiplied with the function obtained from interpolation between plasma-to-whole blood ratio values from the manual samples to generate the corresponding whole-plasma curve.

For [^18^F]DPA-714, calibrated IDIFs and input function from continuous sampler were corrected for plasma to whole-blood ratios and metabolites to obtain metabolite corrected plasma input functions. The method for plasma/whole blood correction was the same method as described above for [^18^F]FDG. Additionally, for metabolite corrections, parent fractions measured in the manual plasma samples were fitted to a Hill function [25]. Each individual whole-plasma curve was then multiplied by the corresponding fitted Hill function.

#### Kinetic modelling

For [^18^F]FDG, Patlak linearization [26] (using t* = 30 min) was performed to estimate the net tracer flux *K*_*i*_ (mL·cm^− 3^·min^− 1^) using the 11 different calibrated IDIFs and using a same target tissue time-activity-curve (TAC) from a VOI manually drawn using the EARL2 reconstruction on the striatum. Four patients did not have any high tumor uptake so the striatum was chosen as target region because it is a region with high level uptake similar to that seen in cancers. The *EARL2_AA* IDIF was used as reference because arterial continuous sampler was not available for [^18^F]FDG. Moreover, EARL2 is the standard reconstruction for whole-body and because a previous study concluded that AA is the structure of choice for defining IDIF for [^18^F]FDG [8]. Therefore, the *K*_*i*_ extracted with the 10 others calibrated IDIFs were compared to the *K*_*i*_ extracted with the calibrated *EARL2_AA* IDIF.

For [^18^F]DPA-714, Logan linearization [27] (using t* = 30 min) was performed to estimate the distribution volume (*V*_*T*_) (mL·cm^− 3^) using the 11 different calibrated IDIFs and using a same target tissue time-activity-curve from a VOI manually drawn using the EARL2 reconstruction on the cerebral frontal region. The brain was chosen because post COVID syndrome might be associated with cerebral neuroinflammation on [^18^F]DPA-714 PET [28]. The *V*_*T*_ extracted with the 11 calibrated IDIFs were compared to the *V*_*T*_ extracted with the calibrated input function obtained from an arterial continuous sampler used as reference. The kinetic modelling was performed using in-house developed software.

## Results

### Study population

**Fig. 1:**
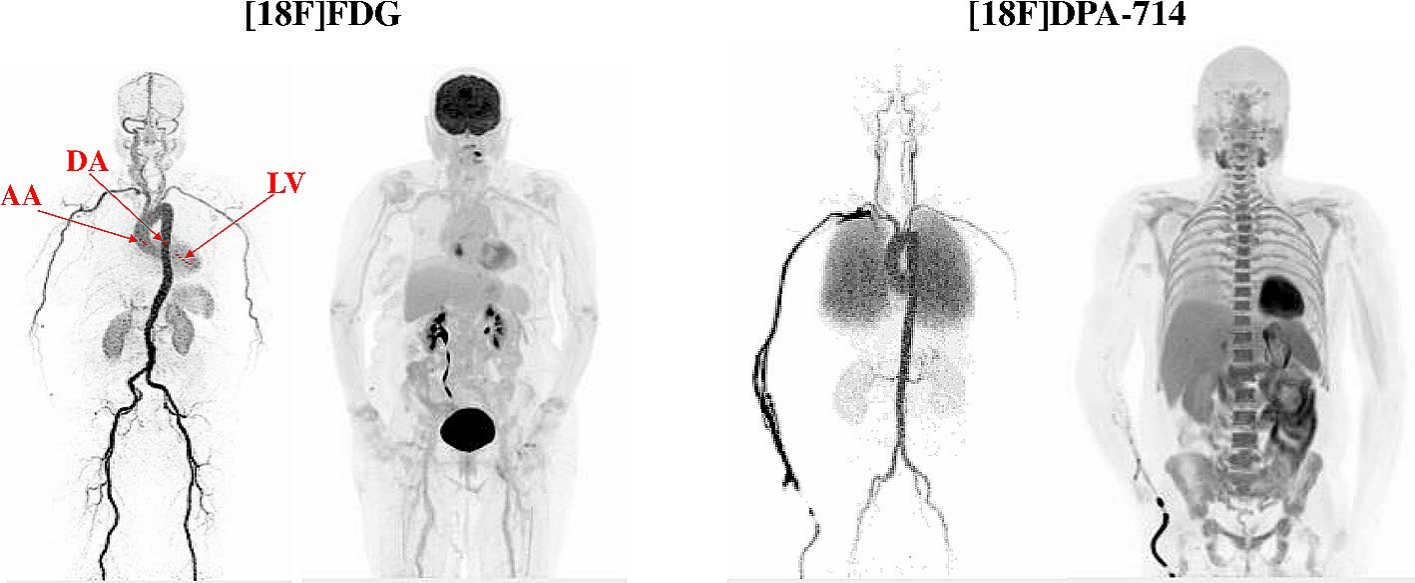
Example of [^18^F]FDG and [^18^F]DPA-714 PET Maximum Intensity Projection images on the 25–30 s post-injection frame with ascending aorta (AA), descending aorta (DA) and left ventricular cavity (LV) volume of interests and the 50–60 min post-injection frame on the EARL2 reconstruction

For [^18^F]FDG, 2 female patients and 6 male patients were included in this study, with a mean age of 62 ± 10 years. For [^18^F]DPA-714, 3 female patients and 7 male patients were included in this study, with a mean age of 49 ± 5 years. Table 2 summarizes the patient characteristics for each tracer. Images of a patient for each tracer are given in Fig. 1.

**Table 2:** Patient demographics (*N* = 18)

Characteristics	[^18^F]FDG (*N* = 8)	[^18^F]DPA-714 (*N* = 10)
Age (years), average (SD)	62 (10)	49 (5)
Gender, n		
Men	6	7
Women	2	3
Weight (kg), average (SD)	85 (18)	97 (28)
Height (cm), average (SD)	180 (13)	178 (10)
Injected dose (MBq), average (SD)	241 (61)	222 (27)

### IDIF validation using calibration assessment with manual blood samples

**Fig. 2:**
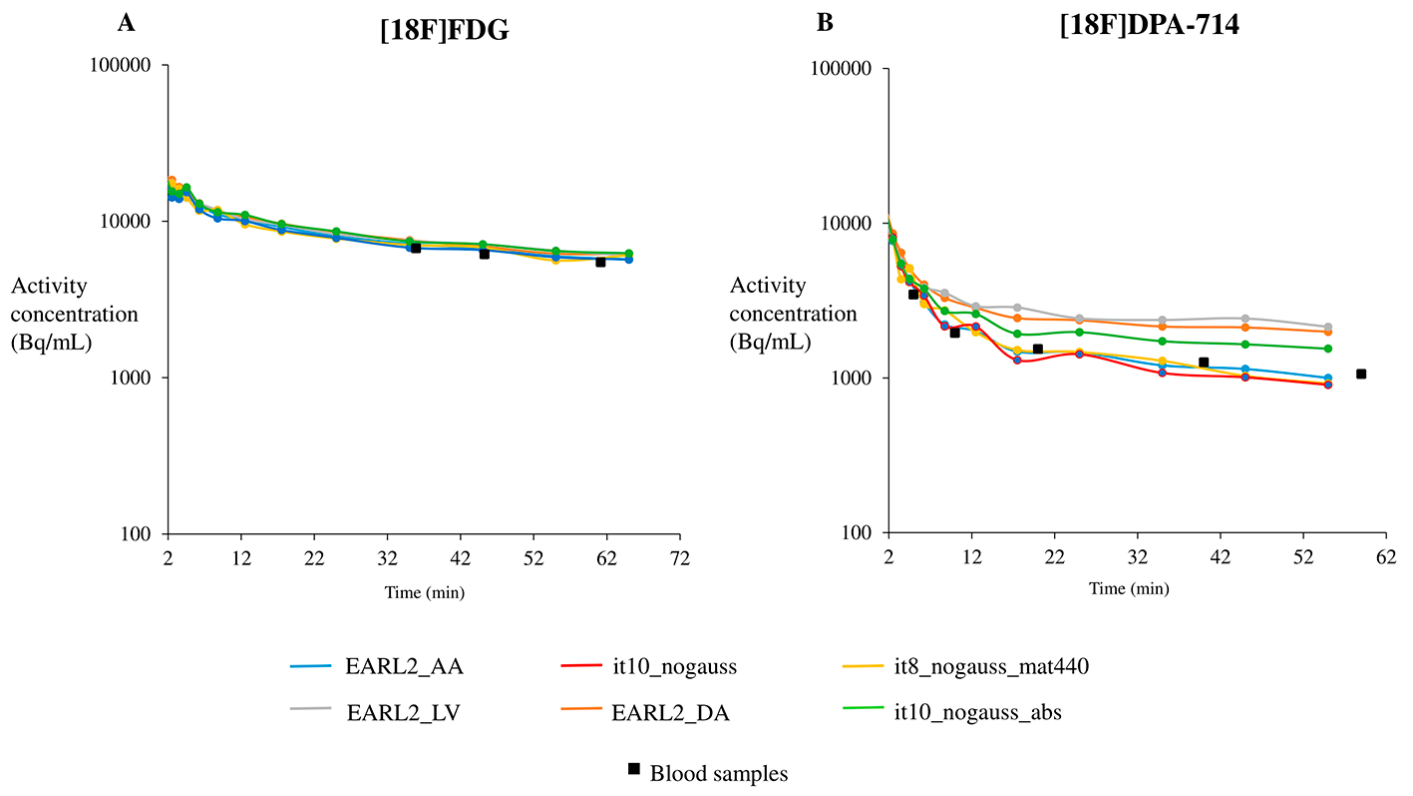
Example of the tail of the time-activity curves from imaging-derived input functions at different locations and different reconstruction settings for [^18^F]FDG (**A**) and for [^18^F]DPA-714 (**B**) (AA: Ascending Aorta, DA: Descending Aorta, LV: Left Ventricular Cavity, it: iterations, gauss: Gaussian post-filtering, mat440: matrix 440 × 440, abs: absolute scatter correction method). All IDIFs were extracted using AA location except for EARL2_DA and EARL2_LV

**Fig. 3:**
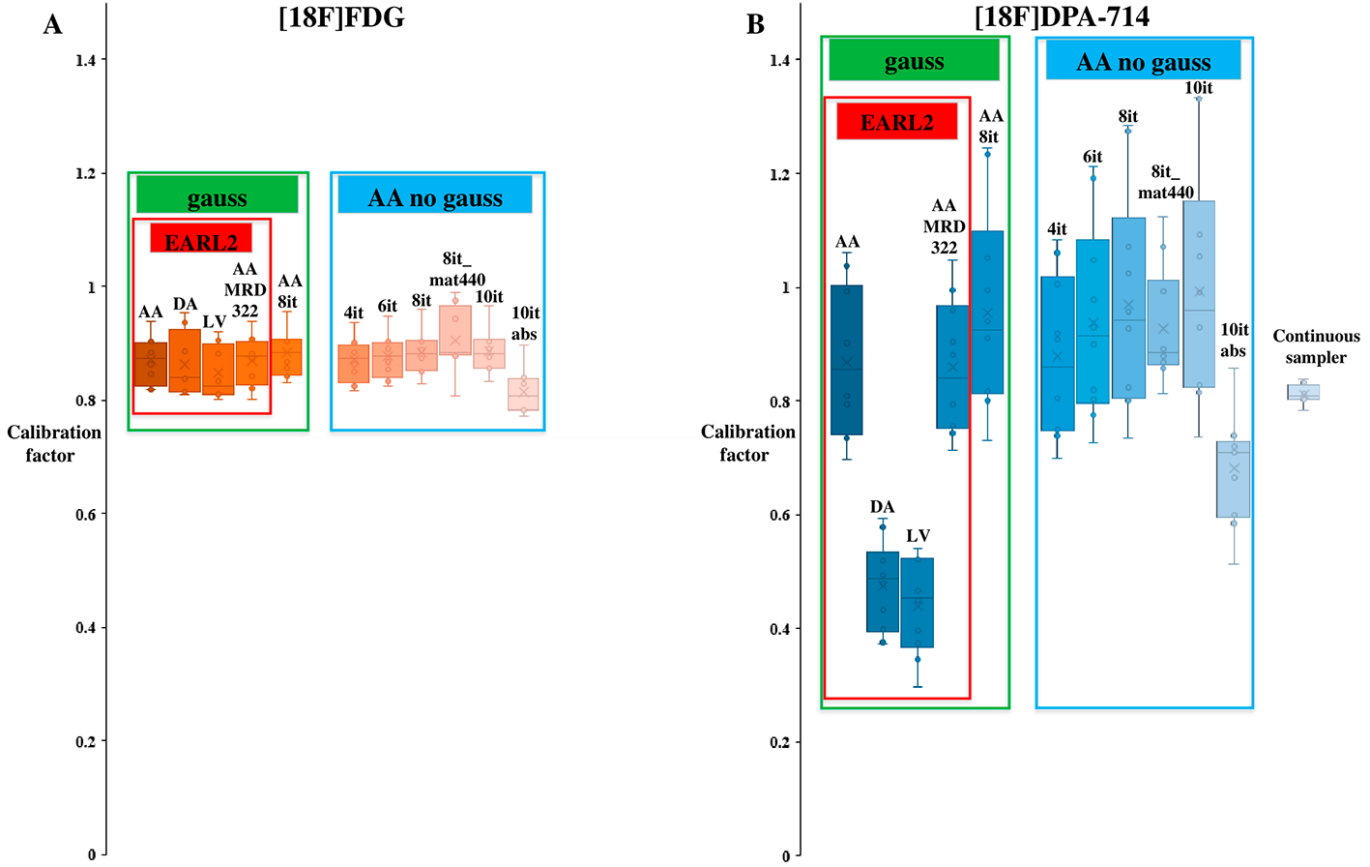
Boxplots illustrating the spread of calibration factors for [^18^F]FDG (**A**) and for [^18^F]DPA-714 (**B**) using the different input functions (gauss: Gaussian post-filtering, AA: Ascending Aorta, DA: Descending Aorta, LV: Left Ventricular Cavity, MRD: maximum ring difference, it: iterations, mat440: matrix 440 × 440, abs: absolute scatter correction method)

For each tracer, examples of IDIF time-activity curves derived at different locations and using different reconstruction settings are displayed in Fig. 2. For [^18^F]FDG, the accuracies and the precisions of estimated IDIF CFs were high (mean calibration factor (CF) ≥ 0.82 & SD ≤ 0.06). Compared to the mean CF using *EARL2_AA* IDIF (0.87), the mean CF value of other IDIFs was similar, e.g. the maximum difference was observed for the mean CF using the *it10_nogauss_abs* IDIF (mean CF = 0.82). For [^18^F]DPA714, the results showed also high accuracy in the estimation of IDIF CFs (mean CF ≥ 0.86) except when using *it10_nogauss_abs*, *EARL2_DA* and *EARL2_LV* (respectively mean CF = 0.68, 0.47 and 0.44). Furthermore, the precision of the different AA IDIFs was low (SD ≥ 0.10). The CF improved using more iterations at the cost of increased variability (mean CF = 0.99 (SD = 0.21) for *it10_nogauss_AA*). The mean CF value using the maximum ring difference (MRD) 322 compared to MRD85 and the mean CF value using the matrix 440 × 440 (high-resolution) compared to the matrix 220 × 220 were similar (respectively difference = -1.1% and − 4.1%). The results of CF are summarized in Table 3; Fig. 3.

**Table 3:** Mean value and standard deviation of the calibration factors using blood samples for the input functions using different locations or reconstruction settings

Input function	[^18^F]FDG (*N* = 8)	[^18^F]DPA-714 (*N* = 10)
Continuous blood sampler	N/A	0.81 (0.02) *
EARL 2_AA	0.87 (0.04)	0.87 (0.13)
EARL 2_DA	0.86 (0.06)	0.47 (0.08)
EARL 2_LV	0.85 (0.05)	0.44 (0.08)
EARL 2_MRD322_AA	0.87 (0.05)	0.86 (0.12)
it8_AA	0.88 (0.04)	0.95 (0.18)
it4_nogauss_AA	0.87 (0.04)	0.88 (0.14)
it6_nogauss_AA	0.88 (0.04)	0.94 (0.17)
it8_nogauss_AA	0.88 (0.04)	0.97 (0.19)
it8_nogauss_mat440_AA	0.91 (0.06)	0.93 (0.10)
it10_nogauss_AA	0.89 (0.04)	0.99 (0.21)
it10_nogauss_abs_AA	0.82 (0.04)	0.68 (0.10)

*Continuous blood sampler was not available for 2 patients (*N* = 8). AA: Ascending Aorta;

DA: Descending Aorta; LV: Left Ventricular Cavity; it: iterations; mat: matrix; gauss: Gaussian post-filtering, MRD: maximum ring differences; abs: absolute scatter correction method

### Uptake ratio between organs and aorta

The mean SUVbw on the 50–60 min post-injection frame of the *AA_EARL2* IDIF was 5.5 times lower for [^18^F]DPA-714 than for [^18^F]FDG. The average liver, lung, myocardium, and spleen uptake ratio between organs and AA were respectively 9.8, 19.5, 8.9, 16.4 times higher for [^18^F]DPA-714 than for [^18^F]FDG. The results are summarized in Table 4.

**Table 4:** Average (SD) SUVbw of organs and blood pool on the 50–60 min post-injection frame on the EARL2 reconstruction and average (SD) SUVbw ratio of organs with blood pool

[^18^F]FDG (N = 8)		[^18^F]DPA-714 (N = 10)	
Ascending aorta (AA)			
2.2 (0.4)		0.4 (0.1)	
Liver	Liver/AA	Liver	Liver/AA
2.7 (0.5)	1.3 (0.1)	4.9 (1.3)	12.8 (2.7)
Lung	Lung/AA	Lung	Lung/AA
0.5 (0.1)	0.2 (0.0)	1.5 (0.7)	3.9 (1.4)
Myocardium	Myocardium/AA	Myocardium	Myocardium/AA
5.8 (3.8)	2.8 (2.0)	9.4 (2.3)	24.5 (6.2)
Spleen	Spleen/AA	Spleen	Spleen/AA
2.4 (0.8)	1.1 (0.4)	7.0 (2.0)	18.0 (2.5)

### Illustration of the impact of different calibrated-IDIFs using kinetic modelling

**Fig. 4:**
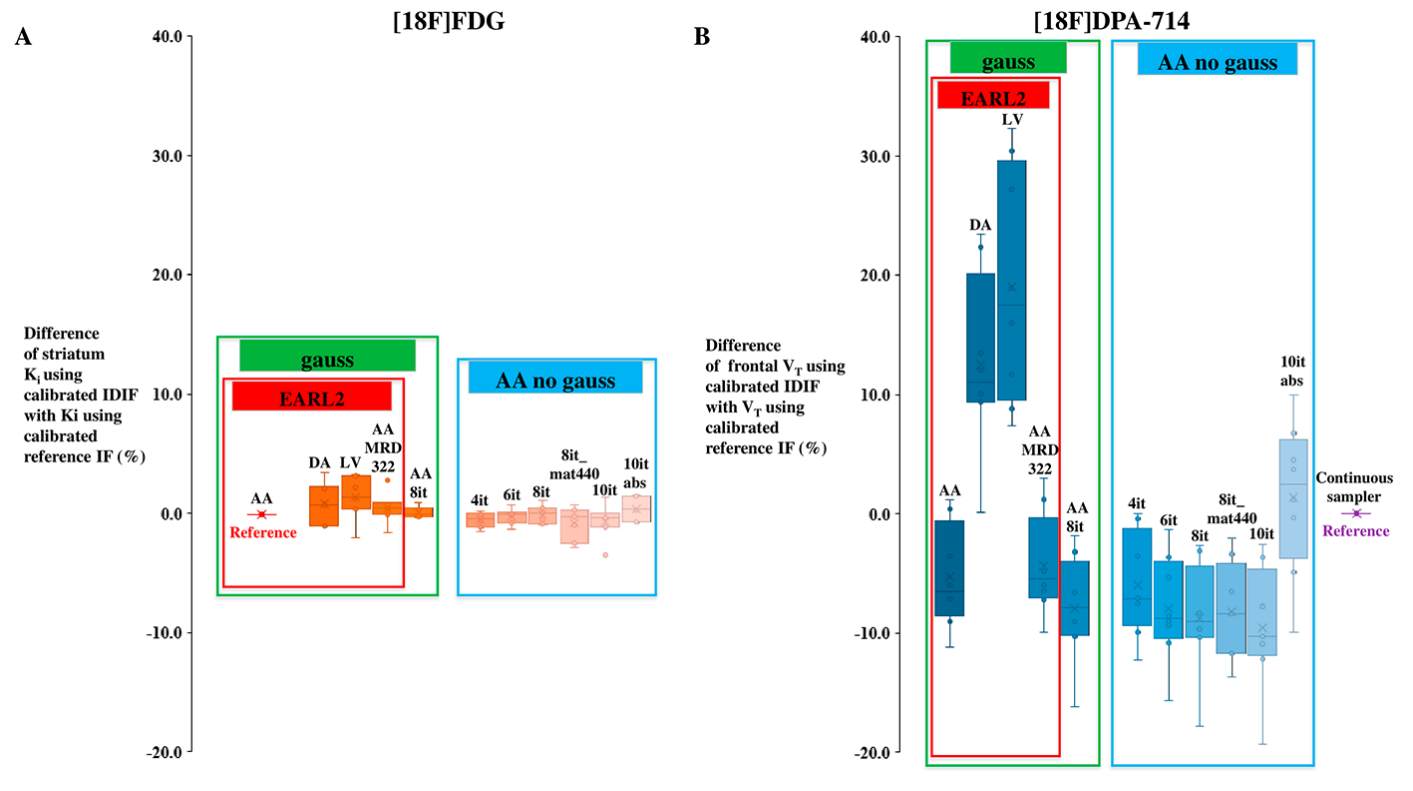
Boxplots illustrating the spread of simplified kinetic parameters values for [^18^F]FDG (**A**) and [^18^F]DPA-714 (**B**) obtained using the different calibrated image-derived input functions compared to the simplified kinetic parameters values obtained using the reference input function (EARL2_AA for [^18^F]FDG and continuous sampler for [^18^F]DPA-714) (gauss: Gaussian post-filtering, AA: Ascending Aorta, DA: Descending Aorta, LV: Left Ventricular Cavity, it: iterations, mat440: matrix 440 × 440, MRD: maximum ring differences, abs: absolute scatter correction method)

**Fig. 5:**
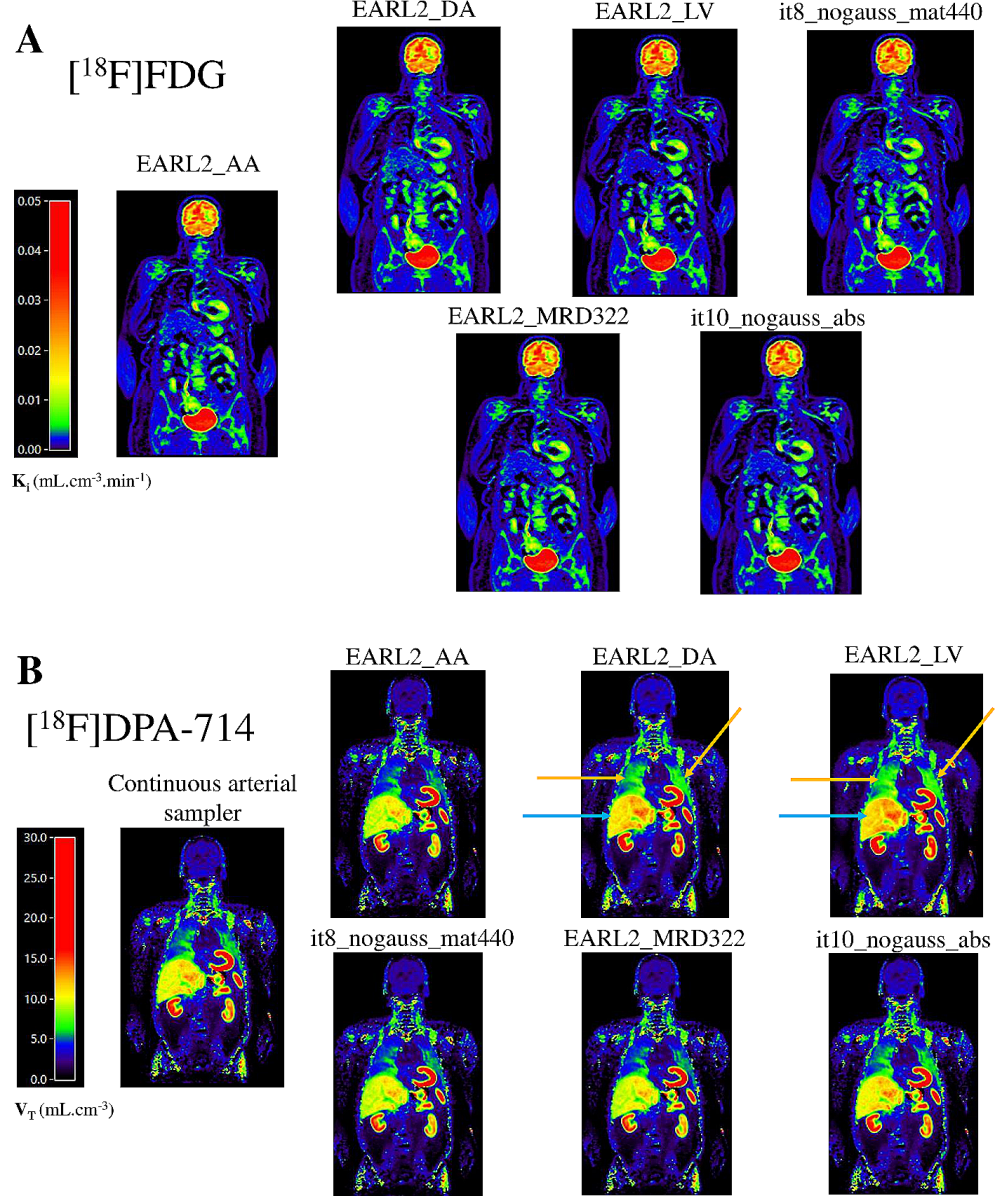
Coronal views of [^18^F]FDG *K*_*i*_ parametric images from Patlak linearization (**A**) and [^18^F]DPA-714 *V*_*T*_ parametric images from Logan linearization (**B**) using different calibrated input functions and the same whole body reconstruction (EARL 2). All IDIFs were extracted using AA location except for EARL2_DA and EARL2_LV. For [^18^F]FDG, no significant visual differences are seen between *K*_*i*_ parametric images using the different IDIFs. For [^18^F]DPA-714, compared to the *V*_*T*_ parametric images obtained with input function using continuous arterial sampler (reference), visual differences are seen using EARL2_DA and EARL2_LV IDIFs, significantly in lungs (orange arrows) and liver (blue arrows) (AA: Ascending Aorta, DA: Descending Aorta, LV: Left Ventricular Cavity, it: iterations, mat440: matrix 440 × 440, MRD: maximum ring differences, abs: absolute scatter correction methods)

For [^18^F]FDG, one patient was excluded for this analysis due to movements of the head during the acquisition. Compared to striatum *K*_*i*_ extracted using calibrated *EARL 2_AA* IDIF as reference, striatum *K*_*i*_ mean differences were < 2% using other calibrated IDIFs. The maximum mean difference was for IDIF using calibrated *EARL 2_LV* (+ 1.6%). For [^18^F]DPA-714, two patients were excluded for this analysis because the arterial continuous sampler acquisition failed. Compared to frontal *V*_*T*_ extracted using calibrated continuous sampler input function as reference, frontal *V*_*T*_ mean differences was low using calibrated AA IDIFs (for examples, *V*_*T*_ mean difference = -5.3% using calibrated *EARL2_AA* IDIF or *V*_*T*_ mean difference = -8.2% using calibrated IDIF with the *it8_nogauss_mat440_AA* reconstruction, which is the high-resolution reconstruction usually applied for brain tracers) but higher using calibrated EARL2_DA and EARL2_LV IDIFs (respectively + 12.5% and + 19.1%). The results of the calibrated-IDIF effect on simplified kinetic parameters are summarized in Table 5; Fig. 4. For both tracers, typical examples of parametric images using Patlak or Logan linearization with different calibrated-IDIF derived at different locations and using different reconstruction settings are displayed in Fig. 5.

**Table 5:** Mean value and standard deviation of the percentage of difference of the simplified kinetic parameters values (striatum *K*_*i*_ using Patlak linearization for [^18^F]FDG and frontal *V*_*T*_ using Logan linearization for [^18^F]DPA-714) extracted from the different calibrated IDIF compared to the simplified kinetic parameters values as reference obtained using calibrated IDIF from EARL2 reconstruction for [^18^F]FDG and using calibrated input function from continuous blood sampler for [^18^F]DPA-714, with the same tissue time-activity curve from EARL 2

	[^18^F]FDG (*N* = 7)*	[^18^F]DPA-714 (*N* = 8)**
Continuous sampler	N/A	Reference
EARL 2_AA	Reference	− 5.3 (4.4)
EARL 2_DA	+1.0 (1.8)	+12.5 (7.5)
EARL 2_LV	+1.6 (1.8)	+19.1 (9.8)
EARL 2_MRD322_AA	+0.7 (1.3)	-4.4 (4.3)
it8_AA	+0.3 (0.5)	-8.0 (4.5)
it4_nogauss_AA	-0.3 (0.6)	-6.0 (4.4)
it6_nogauss_AA	-0.1 (0.7)	-8.0 (4.5)
it8_nogauss_AA	+0.1 (0.8)	-8.8 (4.7)
it8_nogauss_mat440_AA	-0.6 (1.4)	-8.2 (4.1)
it10_nogauss_AA	-0.5 (1.5)	-9.6 (5.2)
it10_nogauss_abs_AA	+0.6 (0.9)	+ 1.4 (6.4)

*Striatum volume of interest was not available for 1 patient due to head movements. **Continuous blood sampler was not available for 2 patients. AA: Ascending Aorta; DA: Descending Aorta; LV: Left Ventricular Cavity; it: iterations; mat: matrix; gauss: Gaussian post-filtering, MRD: maximum ring differences; abs: absolute scatter correction method

## Discussion

This study demonstrates that the accuracy and precision of IDIF obtained with the LAFOV PET/CT system are tracer, reconstruction settings and IDIF VOI locations dependent, which could affect quantitative assessment of kinetic parameters.

Regarding tracer dependency, even if our study showed that the accuracy of AA IDIFs was high and similar for both tracers, the precision of IDIF varies according to the tracer. For [^18^F]FDG, the precision of IDIFs was high so IDIF does not need calibration using manual blood samples, confirming the results already published for [^18^F]FDG using short AFOV PET scanners [11]. For [^18^F]DPA-714, the precision of IDIF was lower suggesting that IDIF needs to be calibrated using a few manual blood samples. Additionally, in case of [^18^F]DPA-714, manual blood samples are also needed for plasma to whole-blood and metabolite corrections. The lower precision could have several explanations. Firstly, we noticed that [^18^F]DPA-714 blood clearance is higher than for [^18^F]FDG, leading to lower count statistics in the blood pool. PET regions with lower counts are more sensitive to the noise than PET regions with higher counts. This also explains why the precision of IDIF was even lower using more iterations for [^18^F]DPA-714 in our study, as more iterations improved convergence but at the cost of increased image noise. Secondly, we showed that contrasts in tracer uptake among organs neighboring the aorta are much higher for [^18^F]DPA-714 than for [^18^F]FDG, due to lower count statistics in the blood pool for [^18^F]DPA-714 but also due to the clearly higher liver, lung, myocardium and spleen uptakes for [^18^F]DPA-714. These higher contrasts lead to bias from the scanners scatter correction algorithm [29]. As reported previously, the accuracy is lower as the tail regions have comparatively low counts using the relative scatter correction method [30]. Thirdly, bias on manual arterial samples used for calibration could also explain the CF variability of IDIF. However, the precision of the whole blood IF from the continuous sampler was high in our study using the same manual arterial samples for calibration. Using calibrated AA IDIFs, our results exhibited similar frontal *V*_*T*_ values compared to frontal *V*_*T*_ values extracted using calibrated continuous sampler input function, so AA IDIF can replace continuous sampler for quantification with Logan graphical method after calibration against manual arterial samples. However, for [^18^F]DPA-714 quantification using non-linear method, further validation against continuous blood sampling is needed because it is more sensitive to the initial part of the input function than linear method. Linear method for macroparameter estimation is based on the whole area under the curve (AUC) of the input function in which the peak contributes very little [31] whereas microparameters estimation using non-linear method needs stringent requirements of the initial part of the input function. LAFOV PET/CT system may improve microparameters estimation because of increased sensitivity leading to better capturing rapid kinetics of the tracer in the blood compartment and the multiple organs.

Regarding IDIF location for [^18^F]FDG, our results showed similar CF for AA, DA or LV IDIFs. Using the LAFOV Siemens Biograph Vision Quadra, Sari et al. showed also good agreement between the IDIFs derived from AA, DA, LV, and left atrium. However, authors exhibited that AUC derived from LV had higher relative standard deviation than AUC derived from the other IDIF regions suggesting worse precision of the estimated blood pool [3]. Using the LAFOV uEXPLORER, Zhang et al. exhibited in a figure a similar peak amplitude between IDIF derived from DA and IDIF derived from LV but a smoother and lower tail of the IDIF derived from DA compared to the IDIF derived from LV [32]. A previous study which evaluated the accuracy of IDIF from AA, LV, and abdominal aorta IDIFs in 136 [^18^F]FDG PET/CT scans including patients with various cancers showed that the metabolic rate of glucose (MRGlu) of lesions using AA IDIF had the strongest correlation with the MRGlu using arterial sampling [11]. However, this strong relationship was similar for all IDIFs. Van der Weerdt et al. showed a better accuracy for AA and DA than LV and left atrium IDIFs and a better precision for AA than DA IDIF in 18 cardiac [^18^F]FDG PET scans with arterial samplings [8]. The higher accuracy differences between AA and LV compared to our report could be due to spillover effect linked to higher [^18^F]FDG uptake in myocardium in the cardiac study. Indeed, in the cardiac study, patients had a light breakfast before [^18^F]FDG injection which induces endogenous insulin secretion and then high myocardium uptake. In our study, patients with oncological diseases were fasting at least 6 h so the myocardium uptake was probably lower than in the cardiac study. This effect comes from high uptake regions close to IDIF regions because of the limited spatial resolution of PET scanners and movements. Since those reports, spatial resolution of PET scanners has improved substantially and could be also a reason why we found lower differences between AA and LV IDIFs for [^18^F]FDG. For [^18^F]DPA-714, our study exhibited that the accuracy of DA and LV IDIFs were lower than the accuracy of AA IDIFs. Due to the small size of the VOI, IDIFs potentially could suffer from partial-volume effect. However, the size of the 3 different IDIFs VOIs was the same. The main factor of this lower accuracy for DA IDIF and LV IDIF is probably the spillover effect. The reason why LV and DA IDIFs are more sensitive to this effect for [^18^F]DPA-714 than for [^18^F]FDG is due to the respectively markedly higher [^18^F]DPA-714 myocardium and posterior lung uptakes than [^18^F]FDG.

Regarding reconstruction settings, for [^18^F]DPA-714, our results exhibited that CF improved using more iterations because more iterations improves the contrast in the images [33]. We observed that the variability also increased using more iterations. This lower precision is due to higher image noise using more iterations [34]. Our study showed a significant lower IDIF accuracy for the absolute scatter correction method compared to the relative scatter correction set as default. Our results are consistent with a previous study showing an underestimation of scatter using the absolute scatter correction method [29]. However, the absolute scatter correction method improved the precision of IDIF in our study. There are two different methods currently available to perform scatter scaling of the most widely used scatter correction method algorithm, based on the single scatter simulation (SSS) [35]: the absolute method which utilizes the SSS scatter distribution directly and the relative method. The relative method is the default technique implemented in clinical PET imaging because it compensates for multiple scatter and for scatter contributions, scaling the activity located outside the axial FOV. The higher contrast between normophysiological uptakes for [^18^F]DPA-714 than for [^18^F]FDG probably explains why differences were greater for the CF IDIF between the use of relative and absolute scatter correction for [^18^F]DPA-714 compared to [^18^F]FDG data, which is consistent with two previous studies [29, 36].

We hypothesize that the accuracy of IDIF for tracers such as [^18^F]DPA-714 with high uptake in the main organs close to the aorta could sometimes also be low due to high variability of IDIF and need manual blood samples for calibration contrary to tracers such as [^18^F]FDG with low contrast. Moreover, for tracers such as [^18^F]DPA-714 with high myocardium and/or posterior lung uptake, DA and/or LV IDIFs are not accurate due to the spillover effect. Therefore, we conclude that the use of IDIF has to be validated for each tracer using LAFOV PET/CT system.

## Conclusion

For [^18^F]FDG, IDIF do not need calibration against manual blood samples. For [^18^F]DPA-714, AA IDIF can replace the continuous arterial sampling for simplified kinetic quantification but only with calibration against manual arterial blood samples. Findings from this study suggest that the accuracy and precision of IDIF obtained using a LAFOV PET/CT system depend on tracer, reconstruction settings and IDIF location. Therefore, the use of IDIF needs to be validated for each tracer and optimization is warranted.

## Data Availability

The datasets used and/or analyzed during the current study are available from the corresponding author on reasonable request.
